# Gingival Crevicular Fluid Levels of Parathyroid Hormone-Related Protein, RANK Ligand, and Osteoprotegerin in Periodontal Health, Gingivitis, and Periodontitis: A Cross-Sectional Study

**DOI:** 10.3390/jcm15197752

**Published:** 2026-10-08

**Authors:** Bilge Meracı Yıldıran, Nuray Ercan, Esra Ateş Yıldırım, Zeynep Akgül, Özgür Mehmet Yis

**Affiliations:** 1 Department of Periodontology, Faculty of Dentistry, Bolu Abant Izzet Baysal University, 14030 Bolu, Türkiye; nuray.ercan@ibu.edu.tr (N.E.); esraates@ibu.edu.tr (E.A.Y.); zeynepakgul@ibu.edu.tr (Z.A.); 2 Department of Medical Biochemistry, Faculty of Medicine, Bolu Abant Izzet Baysal University, 14030 Bolu, Türkiye; ozgurmehmetyis@ibu.edu.tr

**Keywords:** parathyroid hormone-related protein, RANK ligand, osteoprotegerin, periodontal attachment loss, periodontitis

## Abstract

**Background/Objectives**: Parathyroid hormone-related protein (PTHrP) modulates the receptor activator of nuclear factor kappa B ligand (RANKL)/osteoprotegerin (OPG) system in periodontal cells in vitro but has not been measured in gingival crevicular fluid (GCF). This study compared GCF PTHrP, RANKL, and OPG across periodontal health, gingivitis, and periodontitis. **Methods**: This cross-sectional study included 81 participants with periodontal health, gingivitis, or stage III grade B periodontitis (*n* = 27 each). GCF was collected from two interproximal sites per participant. PTHrP, RANKL, and OPG were quantified by ELISA and expressed as total amount per site and concentration. Group comparisons were repeated with adjustment for age and sex. **Results**: PTHrP total amount differed between all three groups, with values of 4.26 ± 2.96, 12.45 ± 5.77, and 19.67 ± 4.59 pg/site in periodontal health, gingivitis, and periodontitis, respectively (*p* < 0.001). RANKL total amount did not differ among groups (median: 51.40, 51.40, and 51.78 pg/site; *p* = 0.977). OPG total amount was lower (median: 53.36, 46.70, and 46.70 pg/site; *p* < 0.001) and the RANKL/OPG ratio higher (median: 0.98, 1.10, and 1.14; *p* < 0.001) in both disease groups than in periodontal health. After adjustment, PTHrP and OPG differences persisted, whereas no pairwise difference in the RANKL/OPG ratio remained significant. PTHrP correlated negatively with OPG (*r* = −0.530) and positively with the RANKL/OPG ratio (*r* = 0.481; both adjusted *p* < 0.001). **Conclusions**: GCF PTHrP total amount differed between periodontal health, gingivitis, and periodontitis, with the highest values in periodontitis. These cross-sectional findings identify an association between GCF PTHrP and periodontal status but do not establish a causal role for PTHrP in periodontal tissue breakdown.

## 1. Introduction

Plaque-induced periodontal diseases comprise gingivitis, an inflammatory condition confined to the gingiva, and periodontitis, in which inflammation extends to the deeper supporting tissues and results in loss of periodontal attachment and alveolar bone [1,2]. According to the Global Burden of Disease Study 2021, the age-standardized prevalence of severe periodontitis was 12,498.3 per 100,000 population [3], and the number of affected individuals is projected to exceed 1.5 billion by 2050 [4]. The disease is initiated by a dysbiotic subgingival biofilm, yet the destruction of the tooth-supporting apparatus is attributed to the host immune and inflammatory response rather than to a direct action of bacteria on bone [2,5]. This host response involves a network of immune, epithelial, and stromal cells, with cytokine and chemokine signaling contributing to inflammatory cell recruitment in periodontal tissues [6]. Longitudinal gingival crevicular fluid (GCF) analyses have identified distinct inflammatory mediator profiles at progressing and stable sites and documented changes in these profiles following periodontal treatment [7]. Periodontal treatment has also been associated with reductions in selected circulating inflammatory markers, although serum biomarker levels were not associated with disease progression in the same investigation [8]. Together, these findings support investigating GCF biomarkers within the broader context of the host inflammatory response, including mediators linking inflammation to periodontal bone loss.

A key link between inflammation and alveolar bone loss is the differentiation and activation of osteoclasts [5]. These processes are regulated by the balance between receptor activator of nuclear factor kappa B ligand (RANKL) and osteoprotegerin (OPG), a soluble decoy receptor that binds RANKL and prevents its interaction with receptor activator of nuclear factor kappa B (RANK) on osteoclast precursors [9]. The relevance of this axis to the periodontium has been supported experimentally, since stimulation of human CD4-positive T cells by Aggregatibacter actinomycetemcomitans induced RANKL production, and administration of OPG diminished alveolar bone destruction and reduced the number of periodontal osteoclasts after microbial challenge in vivo [10]. Evidence assembled from clinical studies of gingival tissue [11], GCF [12], saliva [13], and serum [14] indicates that RANKL is upregulated and OPG is downregulated in periodontitis relative to periodontal health, so that the RANKL/OPG ratio rather than either molecule alone has been proposed as the more informative indicator of the disease [15]. Within GCF specifically, RANKL and OPG have been reported to be differentially expressed in patients with periodontitis [12]. In a study comparing periodontal health, gingivitis, and periodontitis, GCF RANKL remained low in both health and gingivitis but was elevated in periodontitis, whereas OPG was already reduced in gingivitis, with the result that the RANKL/OPG ratio was raised only in periodontitis and correlated positively with probing pocket depth and clinical attachment level (CAL) [16]. Consequently, mediators that modulate the RANKL/OPG balance in periodontal tissues are of interest in this context.

Parathyroid hormone-related protein (PTHrP) is expressed in many normal tissues, where it acts locally rather than as a circulating hormone and signals mainly through the parathyroid hormone (PTH)/PTHrP type 1 receptor (PTH1R) [17,18]. PTHrP has also been identified as an inducible component of the inflammatory cytokine network [18]. Interleukin-1β (IL-1β) and tumor necrosis factor-α (TNF-α) upregulate PTHrP expression, and PTHrP in turn induces proinflammatory cytokines and chemokines through nuclear factor-κB [19,20]. This has been examined in rheumatoid arthritis, a chronic inflammatory disease with periarticular bone destruction, in which secretion of amino-terminal PTHrP was higher in rheumatoid than in osteoarthritic synovial tissue and was stimulated by TNF-α and IL-1β in cultured synoviocytes [21]. Synovial fluid C-terminal PTHrP correlated with C-reactive protein and erythrocyte sedimentation rate, whereas circulating levels did not differ between groups [22]. The effect of PTHrP on bone metabolism depends on the pattern of exposure [18]. Intermittent administration of abaloparatide, a synthetic analog of human PTHrP (1–34), is anabolic and is approved for the treatment of osteoporosis [23], whereas continuous infusion of the same peptide lowered bone mineral density and produced net resorption in mice [24]. In cells of the periodontal attachment apparatus, PTHrP induced RANKL messenger RNA (mRNA) expression and promoted osteoclast differentiation in co-cultures of human periodontal ligament cells and mouse spleen cells, although the reduction in OPG expression did not reach statistical significance [25]. In murine cementoblasts, PTHrP lowered OPG while raising cell-associated RANKL [26]. PTHrP is required for tooth eruption [27] and restrains cementoblast-mediated mineralization in vitro [28]. Under mechanical loading, cyclic tensile strain upregulated PTHrP and PTH1R in cementoblasts [29], local delivery of PTHrP accelerated orthodontic tooth movement in rats [30], and PTHrP has been measured in whole saliva during orthodontic treatment [31]. In experimental periodontal bone loss, intermittent PTHrP (1–34) reduced alveolar bone loss in diabetic rats [32], and abaloparatide protected alveolar bone in ligature-induced periodontitis [33]. Although exogenous PTHrP has been shown to preserve alveolar bone in these models, the role of PTHrP in the pathogenesis of periodontal disease remains unclear.

Experimental evidence that PTHrP modulates the RANKL/OPG system in periodontal cells provides a rationale for examining associations among these biomarkers in GCF. This cross-sectional study quantified PTHrP, RANKL, and OPG in the GCF of patients with periodontal health, gingivitis, and stage III grade B periodontitis. GCF PTHrP total amount was the primary outcome. GCF RANKL and OPG total amounts and the derived RANKL/OPG ratio were secondary outcomes. Associations of GCF PTHrP with the RANKL/OPG ratio and with clinical periodontal parameters were examined as secondary analyses. We hypothesized that GCF PTHrP and the RANKL/OPG ratio would be higher in periodontitis than in either gingivitis or periodontal health.

## 2. Materials and Methods

### 2.1. Study Design

For this cross-sectional comparative study, individuals presenting to the periodontology clinic at the Faculty of Dentistry, Bolu Abant Izzet Baysal University, were recruited. Approval for the protocol was granted by the Non-Interventional Clinical Research Ethics Committee of Bolu Abant Izzet Baysal University (Approval No: 2026/236, Date: 20 May 2026) and was registered at ClinicalTrials.gov (NCT07793370). Every procedure complied with the Declaration of Helsinki. Participants were informed about the study verbally and in writing, and each then signed a consent form. The study was designed and reported in accordance with the Strengthening the Reporting of Observational Studies in Epidemiology (STROBE) statement.

### 2.2. Study Population

**Figure 1:**
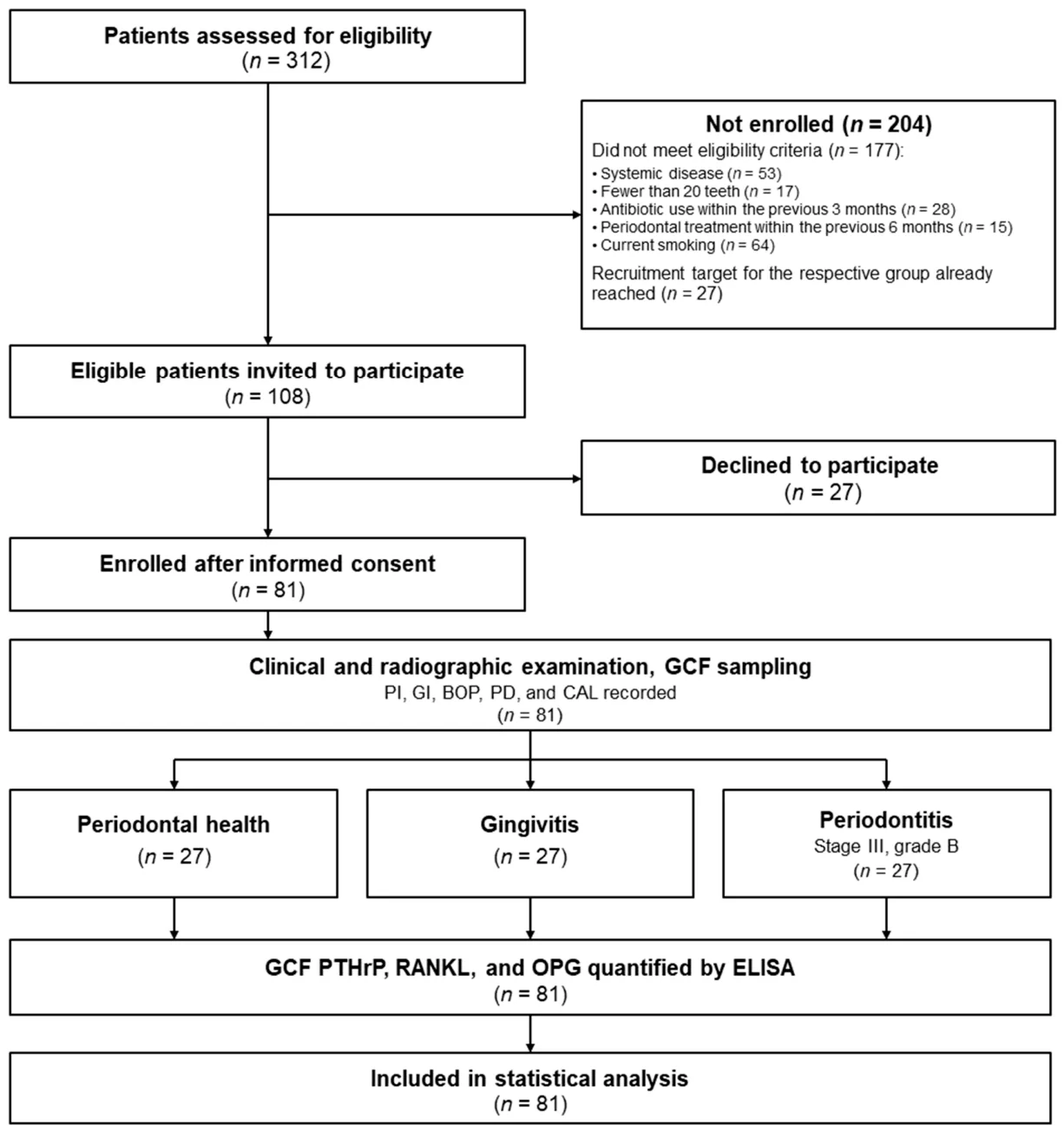
Participant selection and classification according to periodontal status.

Systemically healthy individuals who consecutively attended the Department of Periodontology for periodontal treatment between 1 June and 24 July 2026 were screened for eligibility. Recruitment continued until 27 participants had been enrolled in each periodontal category: periodontal health, gingivitis, and periodontitis (Figure 1).

Eligible participants were ≥18 years of age, had at least 20 natural teeth excluding third molars, and had no systemic conditions known to influence periodontal tissues or bone metabolism, including cardiovascular diseases, atherosclerosis, diabetes mellitus, immune disorders, rheumatoid arthritis, obesity, malignancies, osteoporosis, hyperparathyroidism, and chronic kidney disease. Additional inclusion criteria were no antibiotic use within the previous 3 months, no use of medications known to affect periodontal tissues or bone metabolism, and no history of communicable diseases such as human immunodeficiency virus (HIV), hepatitis B virus (HBV), or tuberculosis. Individuals who were pregnant or lactating, had received periodontal treatment within the previous 6 months, or were current smokers were excluded.

Several measures were implemented to minimize bias. Consecutive attendees were screened using predefined eligibility criteria, and all clinical examinations were performed by a single calibrated examiner. GCF samples were collected according to a standardized protocol by an investigator who was not informed of the participants’ periodontal diagnosis, and laboratory personnel were blinded to participants’ periodontal status. To reduce confounding, current smokers and individuals with systemic conditions or taking medications known to affect periodontal tissues or bone metabolism were excluded.

### 2.3. Periodontal Clinical Examination

One calibrated examiner (B.M.Y.) carried out every clinical examination with a manual Williams periodontal probe (Hu-Friedy, Chicago, IL, USA). Before the study, intra-examiner calibration was performed in five randomly selected patients with periodontitis. Full-mouth probing depth (PD), CAL, and gingival index (GI) were recorded twice, 24 h apart. Reproducibility of PD and CAL was assessed using intraclass correlation coefficients (ICCs; two-way random-effects model, absolute agreement, single measures), while GI agreement was assessed using Cohen’s kappa. Across 804 sites, ICCs were 0.990 (95% CI: 0.988–0.991) for PD and 0.987 (95% CI: 0.984–0.989) for CAL. For GI, Cohen’s κ was 0.960 (95% CI: 0.939–0.980) across 536 sites.

Participants were classified into three groups according to the 2017 World Workshop Classification of Periodontal and Peri-Implant Diseases and Conditions [2,34]. An intact, clinically noninflamed periodontium with PD no greater than 3 mm, bleeding on probing (BOP) at under 10% of sites, and neither interproximal CAL nor radiographic bone loss (RBL) defined periodontal health [34]. Gingivitis was defined as PD ≤ 3 mm and BOP at ≥30% of sites, without interproximal CAL or RBL [34]. Stage III grade B periodontitis required interproximal CAL of at least 5 mm, PD reaching at least 6 mm, and no more than four teeth lost for periodontal reasons [2]. Before interproximal attachment loss was attributed to periodontitis, non-periodontal causes were excluded, including endodontic lesions draining through the marginal periodontium, cervical caries, traumatic gingival recession, and bone loss distal to an extracted third molar.

Four clinical periodontal parameters were recorded: GI, plaque index (PI), PD, and CAL. GI and PI were assessed at four sites per tooth (buccal, lingual/palatal, mesial, and distal) [35,36]. PD and CAL were measured at six sites per tooth (mesiobuccal, mid-buccal, distobuccal, mesiolingual/palatal, mid-lingual/palatal, and distolingual/palatal). CAL was defined as the distance from the cementoenamel junction to the base of the periodontal pocket. Panoramic radiographs obtained for routine diagnostic purposes were used solely to support periodontal classification. RBL was visually estimated at the most affected tooth as a percentage of root length to inform staging, and the bone loss percentage-to-age ratio was used for grading [2]. No linear measurements or repeated assessments were performed. Two periodontists (B.M.Y. and N.E.) jointly evaluated the radiographs, and borderline cases were resolved by consensus. BOP was used only as a participant-level diagnostic criterion for group classification and was not included in the statistical analyses.

### 2.4. Gingival Crevicular Fluid Sampling

Gingival crevicular fluid samples were collected from the mesiobuccal or distobuccal interproximal sites of two non-adjacent single-rooted teeth in each participant. The teeth selected for sampling were not standardized across participants; sampling sites were selected to reflect the defining clinical features of each periodontal group. In the periodontal health group, sites with GI < 1 and PD ≤ 3 mm were sampled. In the gingivitis group, sites with GI ≥ 2 and PD ≤ 3 mm were selected. In the periodontitis group, samples were obtained from the deepest periodontal pockets with PD ≥ 5 mm and CAL ≥ 5 mm. Sampling sites were designated by the clinical examiner, and the investigator who collected the GCF samples was not informed of the participants’ periodontal diagnosis.

Each sampling site was cleared of supragingival plaque, isolated with cotton rolls, and gently air-dried. A standardized paper strip (PerioPaper; Oraflow Inc., Plainview, NY, USA) was inserted into the gingival crevice until slight resistance was felt and left in place for 30 s. Strips visibly contaminated with blood or saliva were discarded. GCF volume was measured using a calibrated Periotron 8010 (Oraflow Inc., Plainview, NY, USA). Each strip reading was converted to GCF volume (µL) using the manufacturer’s calibration curve, and the volumes of the two strips were summed. The two strips collected from each participant were pooled in a single microcentrifuge tube and stored at −80 °C until biochemical analysis. Biomarkers were measured in the pooled samples, with the participant as the unit of analysis.

### 2.5. Biochemical Analysis

GCF levels of PTHrP, RANKL, and OPG were determined using commercially available enzyme-linked immunosorbent assay (ELISA) kits according to the manufacturers’ protocols. All analyses were performed at the Department of Biochemistry, Faculty of Medicine, Bolu Abant Izzet Baysal University. Laboratory personnel performing assays were blinded to the clinical status of the participants.

For each participant, pooled GCF samples were eluted from the paper strips in 400 μL phosphate-buffered saline (PBS) with shaking at room temperature for 30 min, followed by centrifugation at 5800 rpm for 10 min [37]. The supernatant was collected and used for biochemical analyses.

PTHrP was measured using a Human PTHLH/PTHrP Sandwich ELISA Kit (LS-F22967, LSBio, Shirley, MA, USA; detection range: 15.63–1000 pg/mL; sensitivity, <9.38 pg/mL). According to the manufacturer, both the capture and detection antibodies are rabbit polyclonal antibodies raised against human PTHrP (amino acids 37–177; UniProt P12272), although their exact binding epitopes were not specified. The standard is native full-length human PTHrP purified from human plasma. The assay measures immunoreactive PTHrP without distinguishing intact protein from processed fragments. No detectable cross-reactivity with human PTH (1–84) was reported under the conditions tested. Throughout this article, PTHrP refers to immunoreactive PTHrP measured by this assay.

Briefly, 100 μL of standards, blanks, or samples were added to antibody-precoated wells and incubated for 90 min at 37 °C. After aspiration, 100 μL of biotinylated detection antibody was added and incubated for 1 h at 37 °C. The wells were washed three times, followed by addition of 100 μL horseradish peroxidase (HRP) conjugate and incubation for 30 min at 37 °C. After five additional washing cycles, 90 μL 3,3′,5,5′-tetramethylbenzidine (TMB) substrate was added and incubated for 15 min at 37 °C protected from light. The reaction was terminated with 50 μL stop solution.

RANKL was measured using a Human TNFSF11/RANKL/TRANCE Sandwich ELISA Kit (LS-F24426, LSBio, Shirley, MA, USA; detection range: 78–5000 pg/mL; sensitivity, <10 pg/mL). Briefly, 100 μL of standards, blanks, or samples were incubated in antibody-precoated wells for 90 min at room temperature. The wells were washed three times, and 100 μL biotinylated detection antibody was added and incubated for 1 h at 37 °C. Following three washing cycles, 100 μL HRP-streptavidin conjugate was added and incubated for 45 min at 37 °C. After five additional washes, 100 μL TMB substrate was added and incubated for approximately 15–30 min at 37 °C protected from light. The reaction was stopped by adding 100 μL stop solution.

OPG was measured using a Human OPG/Osteoprotegerin Sandwich ELISA Kit (LS-F5164, LSBio, Shirley, MA, USA; detection range: 0.156–10 ng/mL; sensitivity, <0.059 ng/mL). Briefly, 100 μL of standards, blanks, or samples were incubated in antibody-precoated wells for 1 h at 37 °C. After aspiration, 100 μL Detection Reagent A was added and incubated for 1 h at 37 °C. The wells were washed three times, followed by addition of 100 μL Detection Reagent B and incubation for 30 min at 37 °C. After five additional washing cycles, 90 μL TMB substrate was added and incubated for 10–20 min at 37 °C protected from light. The reaction was terminated with 50 μL stop solution.

For all assays, absorbance was determined immediately at 450 nm using a Bio-Rad Benchmark Plus Microplate Reader (Bio-Rad Laboratories, Hercules, CA, USA). Samples and standards were analyzed in duplicate, and mean absorbance values were used for calculation. After subtraction of the zero-standard absorbance, standard curves were generated using a four-parameter logistic curve-fitting model. Samples were reanalyzed in fresh wells when the coefficient of variation between duplicate measurements exceeded 15%, when the optical density fell outside the standard curve, or when a technical error was detected. The manufacturer reported intra- and inter-assay coefficients of variation of <6.89% and <8.63% for the PTHrP kit and <10% and <12% for the OPG kit; no precision data were provided for the RANKL kit.

Biochemical findings were expressed as both total amount (pg/site) and concentration (pg/μL). The biomarker amount in each pooled sample was calculated by multiplying the concentration obtained from the standard curve by the elution volume (0.4 mL). No additional dilution factor was applied. The pooled amount was divided by two to obtain the total amount, defined as the mean amount recovered per sampled site during the 30 s collection (pg/site). GCF volume per site was calculated by dividing the combined volume of the two sampled sites by two. GCF biomarker concentration (pg/µL) was calculated by dividing the total amount by the GCF volume per site. The RANKL/OPG ratio was calculated for each participant by dividing RANKL total amount by OPG total amount (both in pg/site); because both biomarkers were measured in the same eluate, the ratio does not depend on GCF volume. Total amount was prespecified as the primary measure for all biomarkers, and concentration was reported as a complementary measure. PTHrP total amount was the primary outcome; RANKL and OPG total amounts and the RANKL/OPG ratio were secondary outcomes.

### 2.6. Statistical Analysis

Sample size was estimated a priori for the primary outcome, GCF PTHrP total amount, using G*Power software (version 3.1.9.6; Heinrich Heine University, Düsseldorf, Germany). As no previous studies had measured GCF PTHrP, a large effect size was assumed based on Cohen’s conventions (f = 0.40) [38]. A fixed-effects, omnibus one-way analysis of variance with three groups, a significance level of 0.05, and a statistical power of 85% (1 − β = 0.85) yielded a minimum total sample size of 72 participants (24 per group). To allow for possible sample loss, 27 participants were enrolled in each group (*n* = 81).

Statistical analyses were performed using R software (version 4.5; R Foundation for Statistical Computing, Vienna, Austria). Data normality was assessed using the Kolmogorov–Smirnov and Shapiro–Wilk tests, and homogeneity of variances using Levene’s test. Categorical variables were compared using Fisher’s exact test with Monte Carlo simulation. The effect size of the association between categorical variables was assessed using Cramér’s V, with 95% CIs calculated by bootstrap resampling. Continuous variables were analyzed using one-way analysis of variance (ANOVA) for normally distributed data, or Welch’s ANOVA when variances were unequal, and the Kruskal–Wallis test for non-normally distributed data. When overall group differences were significant, pairwise comparisons were performed using Tukey’s test following ANOVA, the Games–Howell test following Welch’s ANOVA, and Dunn’s test with Bonferroni correction following the Kruskal–Wallis test. Effect sizes for between-group comparisons of quantitative variables were assessed using eta-squared (η^2^) for ANOVA and as rank-based eta-squared (η^2^_H_) for the Kruskal–Wallis test, with 95% CIs reported.

To account for the age difference among the groups, biomarker total amounts and the RANKL/OPG ratio were also compared by analysis of covariance with group and sex as fixed factors, including their interaction, and age as a covariate. Robust estimation with HC3 heteroscedasticity-consistent standard errors or bootstrap resampling (5000 samples) was used when the assumptions of homogeneity of variance or normality of residuals were not met. In a sensitivity analysis, PTHrP total amount was compared among the groups with GCF volume per site as a covariate. Pairwise comparisons of adjusted means were Bonferroni-adjusted.

Associations between continuous variables were assessed using Pearson’s or Spearman’s correlation coefficients, as appropriate. These analyses were considered secondary and exploratory. The *p*-values for the 26 correlations T were adjusted for multiple testing using the Benjamini–Hochberg false discovery rate procedure, with adjusted *p*-values < 0.05 considered statistically significant.

Normally distributed variables are presented as mean ± standard deviation, non-normally distributed variables as median (minimum–maximum) with mean ranks, and categorical variables as frequency and percentage. Statistical significance was set at *p* < 0.05.

## 3. Results

### 3.1. Participant Characteristics

A total of 81 participants were analyzed, with 27 participants in each group (Figure 1). Clinical measurements and biomarker results were available for all participants, with no missing data or samples excluded from the analyses.

Mean age differed significantly among groups (*p* < 0.001), with participants in the periodontitis group being older than those in the periodontal health and gingivitis groups (44.26 ± 8.34 vs. 33.41 ± 8.93 and 30.89 ± 8.15 years, respectively). Sex distribution did not differ significantly among the groups (*p* = 0.284) (Table 1).

### 3.2. Clinical Findings

All clinical periodontal parameters and GCF volume differed significantly among groups (all *p* < 0.001; Table 2). PI and GI increased across periodontal health, gingivitis, and periodontitis, although PI did not differ between the gingivitis and periodontitis groups. PD and CAL were significantly higher in periodontitis than in the other groups. Median GCF volume per site was 0.23 (0.06–0.52), 0.48 (0.20–0.98), and 0.67 (0.39–1.06) µL/site in the periodontal health, gingivitis, and periodontitis groups, respectively, with significant differences between all groups.

### 3.3. Biochemical Findings

GCF biomarker levels are presented in Table 3. PTHrP total amount increased progressively across the groups (4.26 ± 2.96, 12.45 ± 5.77, and 19.67 ± 4.59 pg/site; *p* < 0.001), with significant differences between all groups. PTHrP concentration also differed among groups (*p* = 0.044), although only the periodontal health and periodontitis groups differed significantly. RANKL total amount did not differ among groups (*p* = 0.977), whereas RANKL concentration was significantly lower in both gingivitis and periodontitis than in periodontal health (*p* < 0.001). OPG total amount was lower in both disease groups than in periodontal health, whereas OPG concentration decreased progressively across the three groups (all *p* < 0.001). The RANKL/OPG ratio differed significantly among groups (*p* < 0.001), with higher values in both the periodontitis and gingivitis groups than in the periodontal health group (1.14, 1.10, and 0.98, respectively), while no significant difference was observed between the gingivitis and periodontitis groups.

Covariate-adjusted comparisons are presented in Table 4. After adjustment for age and sex, PTHrP total amount still differed among all three groups (all *p* < 0.001). OPG total amount remained lower in gingivitis and periodontitis than in periodontal health (*p* = 0.002 and *p* = 0.010, respectively), and RANKL total amount did not differ among the groups (*p* = 0.230). For the RANKL/OPG ratio, the overall group difference remained significant (*p* = 0.015), but no pairwise difference reached statistical significance. Age was not significantly associated with any of these outcomes. After adjustment for GCF volume, PTHrP total amount still differed among all three groups (*p* < 0.001).

### 3.4. Correlations

Correlations are summarized in Table 5. PTHrP total amount showed the strongest associations with periodontal parameters, including PI (*r* = 0.663), CAL (*r* = 0.658), GI (*r* = 0.648), PD (*r* = 0.576), and GCF volume (*r* = 0.647; all adjusted *p* < 0.001). It was also negatively correlated with OPG total amount (*r* = −0.530) and positively correlated with the RANKL/OPG ratio (*r* = 0.481; both adjusted *p* < 0.001), but not with RANKL total amount (adjusted *p* = 0.583). OPG total amount was positively correlated with RANKL total amount (*r* = 0.520, adjusted *p* < 0.001) and inversely correlated with PI, GI, and GCF volume (all adjusted *p* ≤ 0.001). RANKL total amount showed no significant correlation with clinical periodontal parameters. The RANKL/OPG ratio was positively correlated with PI, GI, PD, and GCF volume, whereas its correlation with CAL was not significant after adjustment for multiple testing (*r* = 0.229, adjusted *p* = 0.061).

## 4. Discussion

This cross-sectional study compared GCF PTHrP, RANKL, and OPG in individuals with periodontal health, gingivitis, and stage III grade B periodontitis. PTHrP total amount, the primary outcome, increased across the three groups and differed between all of them. RANKL total amount did not differ among the groups, whereas OPG total amount was lower in both gingivitis and periodontitis than in periodontal health. The RANKL/OPG ratio was higher in both disease groups than in periodontal health in unadjusted comparisons, with no difference between gingivitis and periodontitis; after adjustment for age and sex, the overall group difference remained significant, but no pairwise difference did. PTHrP total amount was negatively correlated with OPG total amount and positively correlated with the RANKL/OPG ratio, but not with RANKL total amount. These findings are consistent with the study hypothesis for PTHrP. For the RANKL/OPG ratio the hypothesis was only partially supported in unadjusted comparisons, because the ratio did not differ between gingivitis and periodontitis, and it was not supported after adjustment for age and sex.

Total amount was prespecified as the primary measure because it expresses the quantity of a mediator recovered during a standardized 30 s collection and does not depend on the measurement of small GCF volumes. In earlier GCF studies, values expressed per 30 s sample were more closely related to periodontal status than values expressed as concentration [39,40]. Median GCF volume per site was approximately 2.9-fold higher in periodontitis than in periodontal health; thus, a larger collected volume may yield a greater recovered amount without a proportional increase in concentration. These findings should also be interpreted in the context of site selection: sampling sites reflected the defining clinical features of each group. In the periodontitis group, the deepest eligible pockets were sampled, which may have increased the GCF volume recovered in this group. Because concentration is obtained by dividing the recovered amount by the GCF volume, a larger exudate volume can lower the concentration of a mediator even when its recovered amount is higher. PTHrP total amount differed among all three groups, whereas its concentration differed only between periodontal health and periodontitis. All pairwise differences in PTHrP total amount remained significant after adjustment for GCF volume. Both measures should therefore be interpreted together, as higher recovered amounts alone do not establish increased local production or biological activity.

To our knowledge, PTHrP has not previously been measured in GCF, precluding direct comparison with earlier periodontal studies. The closest available evidence comes from inflamed joint tissues and fluids [21,22]. In synovial fluid, C-terminal PTHrP (109–141) was higher in rheumatoid arthritis than in osteoarthritis and healthy controls and correlated with C-reactive protein and erythrocyte sedimentation rate, whereas plasma concentrations did not differ among the groups [22]. Synovial fluid concentrations were also higher than plasma concentrations in all three groups, including controls. In contrast, N-terminal PTHrP (1–87) measured in the same synovial fluid samples showed a different pattern, with the highest values in osteoarthritis [22]. Evidence from synovial tissue also supports local PTHrP production [21]. Immunoreactive PTHrP (1–86) released from rheumatoid synovial tissue explants was approximately tenfold higher than that released from osteoarthritic tissue. PTHrP and the PTH/PTHrP receptor were expressed in synovial lining cells, fibroblast-like cells within the pannus, and the synovial vasculature, while TNF-α and IL-1β increased PTHrP release from cultured synoviocytes [21]. The authors attributed the absence of a corresponding increase in N-terminal PTHrP in synovial fluid to proteolytic degradation of the peptide within the fluid [21]. Together, these findings suggest that PTHrP may accumulate locally at sites of inflammation without a corresponding increase in the circulation and that the observed pattern may depend on the PTHrP fragment measured. Within the oral cavity, salivary PTHrP measured with an assay directed against the N-terminal region increased over the first 45 days of fixed orthodontic treatment in periodontally healthy adolescents [31]. Although saliva is not directly comparable with GCF and periodontal disease was not investigated, this finding shows that PTHrP can be detected in oral fluid. However, the biological interpretation of PTHrP measurements depends on the molecular forms detected by the assay. In the present study, ELISA measured immunoreactive PTHrP without distinguishing these forms; therefore, the measurements do not establish its biological activity or demonstrate increased local production in periodontal tissues.

Several mechanisms may contribute to the increase in GCF PTHrP, and the present data cannot distinguish among them. First, the inflammatory environment may upregulate local PTHrP expression. In cultured human vascular endothelial cells, TNF-α and IL-1β increased PTHrP release in a time- and dose-dependent manner [41,42]. The same cytokines also increased PTHrP release from cultured synoviocytes [21]. A comparable pattern has been observed in inflamed oral tissue. In normal human dental pulp, PTHrP immunostaining was confined to the odontoblastic and subodontoblastic regions and a few scattered fibroblasts, with no staining in the vascular compartment. In inflamed pulp, PTHrP-positive cells were additionally detected in the pulp stroma and in endothelial and vascular smooth muscle cells [43]. Although that study was descriptive and examined pulp rather than periodontal tissue, it suggests that inflammation may broaden the distribution of PTHrP-expressing cells within dental tissues. Second, the increase may partly reflect exudative changes. GCF is an inflammatory exudate [44], and its volume increased with periodontal disease severity. PTHrP total amount was also positively correlated with GCF volume. Thus, the greater amount of PTHrP recovered in disease may partly reflect increased GCF volume and should not be interpreted as direct evidence of increased local PTHrP production. Third, resident cells of the periodontal attachment apparatus may represent a potential source of PTHrP. Dental follicle progenitor cells and periodontal ligament cells express PTHrP and signal through PTH1R during root formation and tooth eruption [45,46]. Whether this pathway is similarly active during periodontal inflammation remains unknown. Cytokine-induced endothelial PTHrP expression has also been interpreted as a compensatory response [41], so a higher local amount should not be read as evidence of a destructive role. PTHrP expression has not been directly examined in human gingival tissues across periodontal health, gingivitis, and periodontitis. Identifying its cellular sources in these tissues would help to clarify this.

In the present study, PTHrP total amount was negatively correlated with OPG total amount and positively correlated with the RANKL/OPG ratio, but was not correlated with RANKL total amount. Experimental studies have generally shown that PTHrP increases RANKL expression and decreases OPG expression in cells of the tooth and periodontium [26,47,48], and a similar pattern has been reported in bone [49]. These effects have been reported in murine cementoblasts [26], dental follicle and stellate reticulum cells, in which PTHrP neutralization also reduced osteoclast formation [47], human dental follicle cells [48], and a murine osteocyte cell line exposed to full-length PTHrP (1–141) [49]. However, the effect on OPG appears less consistent than that on RANKL. In human periodontal ligament cells, PTHrP induced RANKL mRNA expression, whereas the reduction in OPG did not reach statistical significance [25]. Similarly, in human dental follicle cells, the reduction in OPG was transient, occurring at 0.5 and 1 h before returning toward baseline [48]. Although these studies were conducted predominantly in cell culture, often using murine cells and models of tooth eruption or root formation rather than periodontitis, the inverse association between PTHrP and OPG observed in the present study is broadly consistent with these experimental findings. However, this association does not establish that the immunoreactive PTHrP detected in GCF suppresses OPG production in periodontal tissues.

In contrast, the absence of an association between PTHrP and RANKL differs from most in vitro findings. Several factors may contribute to this discrepancy. First, in vivo responses may not reproduce those observed in vitro. In the same study that reported increased RANKL and decreased OPG in a murine osteocyte cell line, mice with osteocyte-targeted knockdown of PTHrP showed reduced trabecular bone volume and bone formation but no change in osteoclast surface or number [49]. The authors suggested that RANKL induction by osteocyte-derived PTHrP may not be required for physiological regulation of osteoclasts in vivo [49]. Second, the effects of PTHrP on the RANKL/OPG system may be transient [48], whereas a single cross-sectional GCF sample cannot capture temporal changes. Third, different PTHrP fragments may exert different effects. The C-terminal peptide PTHrP (107–111), for example, increased OPG, decreased RANKL, and reduced osteoclast numbers in a rat tibial defect model [50]. Because the assay used in the present study measures total immunoreactive PTHrP, it cannot distinguish molecular forms with potentially different biological effects. Finally, GCF measurements primarily reflect soluble RANKL, whereas RANKL is predominantly expressed as a membrane-bound protein [16]. In the present study, RANKL total amount neither differed among the groups nor correlated with clinical periodontal parameters, which may partly explain the absence of an association with PTHrP.

PTHrP and OPG both varied with periodontal status, and their inverse correlation may therefore reflect their respective associations with disease severity rather than a direct biological interaction. Moreover, the higher unadjusted RANKL/OPG ratio in gingivitis and periodontitis reflected lower OPG rather than higher RANKL, and only the difference in OPG remained significant after adjustment for age and sex. Thus, the present findings support an association between PTHrP and the RANKL/OPG balance but do not provide evidence that this relationship is mediated by increased soluble RANKL.

In animal models, exogenous PTHrP and PTH1R agonists have shown protective effects on alveolar bone. In diabetic rats, subcutaneous PTHrP (1–34) administered for 12 weeks reduced alveolar bone loss [32]. In ligature-induced periodontitis, both abaloparatide and teriparatide reduced alveolar bone loss, with a greater effect observed for abaloparatide [33]. Similarly, in ovariectomized rats with ligature-induced periodontitis, abaloparatide reduced alveolar bone loss and increased OPG together with markers of bone formation [51]. Local submucosal administration of abaloparatide combined with mechanical force also increased alveolar bone thickness through periosteal osteogenesis [52]. In the present study, higher immunoreactive PTHrP amounts were detected in GCF from participants with periodontal disease. This observation cannot be directly compared with the effects of administered PTHrP peptides or PTH1R agonists, as our study measured naturally occurring immunoreactive PTHrP without determining its molecular forms or biological activity. In these animal studies, PTHrP (1–34) or PTH1R agonists were administered at pharmacological doses, generally using intermittent regimens. In contrast, sustained local release of PTHrP from a thermosensitive hydrogel in the rat periodontium increased tartrate-resistant acid phosphatase-positive osteoclasts and the RANKL/OPG ratio and reduced alveolar bone volume and trabecular number [30]. These findings suggest that the effects of administered PTHrP peptides and PTH1R agonists may depend on the administration regimen and experimental setting. This interpretation remains speculative. The temporal pattern of endogenous PTHrP exposure was not assessed in the present study, and pharmacological doses used in animal models cannot be directly compared with the picogram amounts recovered from GCF. Moreover, the present data cannot establish whether higher GCF PTHrP contributes to periodontal tissue destruction, forms part of a reparative response, or simply reflects local inflammatory activity. Two further observations are relevant to a possible destructive role. PTHrP (1–34) reduced gingival IL-1β and IL-6 in the diabetic rat model [32], suggesting that an anti-inflammatory effect cannot be excluded. In addition, the reduction in OPG induced by ligature placement was not reversed by either abaloparatide or teriparatide [33], indicating that the relationship between PTH1R signaling and OPG regulation in vivo may be more complex than suggested by cell culture studies.

Previous GCF studies of RANKL and OPG have yielded inconsistent findings. Two studies reporting concentrations adjusted for GCF volume found higher RANKL, lower OPG, and a higher RANKL/OPG ratio in periodontitis than in periodontal health, one across different severities of periodontitis [12] and the other across periodontal health, gingivitis, and different forms of periodontitis [16]. However, in the former study, RANKL concentration decreased with increasing probing depth within the periodontitis groups [12]. Lower OPG has also been reported in gingivitis. Naturally occurring gingivitis was associated with higher RANKL and lower OPG than periodontal health, whereas experimentally induced gingivitis did not differ from health [53]. Other studies, however, found no significant differences [54,55,56,57]. In a study including mild, moderate, and severe chronic periodontitis and generalized aggressive periodontitis, GCF RANKL, OPG, and the RANKL/OPG ratio did not differ from periodontal health [54]. Similarly, no differences in these markers were observed between periodontally healthy individuals and smokers or nonsmokers with chronic periodontitis [55]. Another study, using the 2017 classification, found no differences in RANKL or the RANKL/OPG ratio among periodontal health, nonsmokers with stage I and II grade A periodontitis, and smokers with stage III grades B and C periodontitis [56]. A site-level analysis showed a different pattern, with higher RANKL total amount at diseased than at clinically healthy sites within the same patients, whereas OPG did not differ; however, sites rather than patients were used as the unit of analysis [57]. Thus, the lower OPG in both disease groups, which persisted after adjustment for age and sex, and the higher unadjusted RANKL/OPG ratio are consistent with part of the available literature. In contrast, the unchanged RANKL total amount differs from studies reporting higher RANKL in periodontal disease.

Several factors may contribute to these inconsistencies. First, the choice of reported measure differs among studies. Because GCF volume increases with periodontal inflammation, total amount and concentration may show different patterns, as illustrated by the study reporting both measures from the same samples [58]. Studies reporting only one measure are therefore not directly comparable. Second, assay sensitivity may influence RANKL measurements. RANKL in control samples was at or below the detection limit in two studies [12,57], was detectable at 46% of control sites compared with 85% of periodontitis sites in another [58], and was detected at only 40–50% of sites in one study reporting no group differences [54]. In the present study, RANKL values were clustered toward the lower end of the assay range, and median RANKL total amounts were nearly identical across the three groups. This limited distribution may have reduced the ability to detect between-group differences in RANKL. Third, the molecular characteristics of RANKL may contribute to variation among studies. RANKL exists in multiple isoforms, and a review of the RANKL/OPG system in periodontal disease suggested that the detection antibodies used in available assays may not adequately recognize all isoforms [15]. Disease activity may also be relevant. RANKL total amount was higher at active than at inactive sites [58], and in a study in which RANKL remained unchanged after scaling and root planing while OPG decreased, the authors attributed this finding partly to possible inactivity of the sampled sites [59]. In the present study, sites were selected according to clinical criteria, and disease activity could not be assessed because of the cross-sectional design.

This study has several limitations. The cross-sectional design precludes conclusions regarding temporal sequence or causality. The observed associations cannot establish whether higher GCF PTHrP precedes periodontal tissue destruction or reflects a response to inflammation or tissue changes. Furthermore, disease activity was not assessed, and the findings do not demonstrate that PTHrP predicts subsequent periodontal breakdown. The sample size calculation assumed a large effect (f = 0.40) with 85% power; therefore, the study may have had limited power to detect small or moderate differences in secondary outcomes. In particular, the nonsignificant difference in RANKL amount per site should not be interpreted as evidence of equivalence among periodontal groups. Age is a potential confounder in this study. Participants with periodontitis were older than those in the other groups, the prevalence of periodontitis increases with age [60], and circulating OPG levels have been reported to vary with age [61]. After adjustment for age and sex, the differences in PTHrP and OPG amounts persisted, whereas the pairwise differences in the RANKL/OPG ratio were no longer significant. Because age and periodontal status were closely related in this sample, residual confounding by age cannot be excluded. Former smoking history and body mass index were not recorded, although current smokers and individuals with obesity were excluded. PTHrP measurements also require cautious interpretation. The assay measures immunoreactive PTHrP but does not identify the molecular forms contributing to the signal. The exact antibody-binding epitopes and the relative detection of individual fragments were not specified by the manufacturer. Therefore, our measurements cannot be attributed to a specific PTHrP fragment or interpreted as evidence of bone-forming or bone-resorbing activity. In addition, the ELISA kits have not been validated for GCF, and dilutional linearity and spike and recovery were not assessed in GCF eluates. Serum PTHrP was not measured; therefore, the relative contributions of local periodontal production and circulating PTHrP to the GCF measurements could not be determined. The detected PTHrP cannot therefore be assumed to originate exclusively from periodontal tissues. GCF total protein was also not measured, precluding normalization to total protein. Because sampling sites were selected to reflect each group’s clinical features, the findings should be interpreted in the context of both participant-level periodontal status and the local characteristics of the sampled sites. Finally, the single-center design may limit the generalizability of the findings.

To our knowledge, these are the first reported GCF PTHrP values across different periodontal conditions and require confirmation in independent populations. Future studies should determine which molecular forms of PTHrP are present in GCF, and examine PTHrP expression in gingival tissues across periodontal health, gingivitis, and periodontitis to identify its potential cellular sources. Longitudinal studies incorporating parallel measurements of GCF and serum PTHrP are also needed to clarify the temporal relationship between PTHrP and periodontal disease and to distinguish local accumulation from contributions of circulating PTHrP.

## 5. Conclusions

PTHrP was measured in GCF across periodontal health, gingivitis, and periodontitis, and its total amount increased progressively across these conditions. In the same samples, RANKL total amount did not differ among the groups, whereas OPG total amount was lower in both disease groups than in periodontal health, also after adjustment for age and sex. The RANKL/OPG ratio was higher in both disease groups in unadjusted comparisons, but no pairwise difference remained after adjustment. These findings indicate an association between GCF PTHrP and periodontal status in this cross-sectional sample. As these are the first reported GCF PTHrP values, they require confirmation in independent populations.

## Figures and Tables

**Table 1:** Demographic characteristics of the study groups.

	Periodontal Health (*n* = 27)	Gingivitis (*n* = 27)	Periodontitis (*n* = 27)	Total (*n* = 81)	Test Statistic	*p*	ES [95% CI]
Age (years)					18.955	**<0.001 ^x^**	0.327 [0.155: 0.457]
Mean ± SD	33.41 ± 8.93 ^a^	30.89 ± 8.15 ^a^	44.26 ± 8.34 ^b^	36.19 ± 10.21			
Median (min:max)	33 (20:52)	32 (20:47)	45 (25:60)	36 (20:60)			
Sex, *n* (%)					2.769	0.284 ^y^	0.187 [0.000: 0.390]
Female	18 (66.7)	16 (59.3)	12 (44.4)	46 (56.8)			
Male	9 (33.3)	11 (40.7)	15 (55.6)	35 (43.2)			

Age is presented as mean ± SD and median (minimum:maximum), and sex as *n* (%). ^x^ One-way ANOVA; ^y^ Monte Carlo corrected Fisher’s exact test. ^a,b^ Within a row, values with different superscript letters differ significantly (*p* < 0.05), whereas values sharing a letter do not. ES [95% CI], effect size with 95% confidence interval: η^2^ for age and Cramér’s V for sex. Bold *p* values indicate statistical significance (*p* < 0.05). SD, standard deviation.

**Table 2:** Clinical periodontal parameters and GCF volume in the study groups.

	Periodontal Health (*n* = 27)	Gingivitis (*n* = 27)	Periodontitis (*n* = 27)	Total (*n* = 81)	Test Statistic	*p*	ES [95% CI]
Plaque index	0.28 (0.13:0.43)/14 ^a^	1.29 (1:2.16)/50.39 ^b^	1.46 (1.05:1.88)/58.61 ^b^	1.19 (0.13:2.16)	55.003	**<0.001 ^x^**	0.680 [0.371: 1.113]
Gingival index	0.31 ± 0.11 ^a^	1.46 ± 0.12 ^b^	1.65 ± 0.27 ^c^	1.14 ± 0.62	790.828	**<0.001 ^y^**	0.919 [0.882: 0.937]
Probing depth (mm)	1.57 (1.24:2.06)/26.39 ^a^	1.63 (1.22:2.51)/28.72 ^a^	3.22 (2.44:4.47)/67.89 ^b^	1.89 (1.22:4.47)	53.045	**<0.001 ^x^**	0.654 [0.352: 1.081]
CAL (mm)	0 (0:0)/27.5 ^a^	0 (0:0)/27.5 ^a^	4.24 (2.95:5.77)/68 ^b^	0 (0:5.77)	75.797	**<0.001 ^x^**	0.946 [0.572: 1.444]
GCF volume per site (µL/site)	0.23 (0.06:0.52)/18.5 ^a^	0.48 (0.20:0.98)/43.02 ^b^	0.67 (0.39:1.06)/61.48 ^c^	0.43 (0.06:1.06)	45.366	**<0.001 ^x^**	0.556 [0.282: 0.955]

Values are median (minimum:maximum)/mean rank, except for the gingival index, which is presented as mean ± SD. ^x^ Kruskal–Wallis H test; ^y^ Welch’s ANOVA. ^a–c^ Within a row, values with different superscript letters differ significantly (*p* < 0.05), whereas values sharing a letter do not. ES [95% CI], effect size with 95% confidence interval: rank-based eta-squared (η^2^_H_) for the Kruskal–Wallis test and eta-squared (η^2^) for Welch’s ANOVA. GCF volume per site is the combined volume of the two sampled sites divided by two. Bold *p* values indicate statistical significance (*p* < 0.05). CAL, clinical attachment level; GCF, gingival crevicular fluid; SD, standard deviation.

**Table 3:** GCF PTHrP, RANKL and OPG levels and the RANKL/OPG ratio in the study groups.

	Periodontal Health (*n* = 27)	Gingivitis (*n* = 27)	Periodontitis (*n* = 27)	Total (*n* = 81)	Test Statistic	*p*	ES [%95 CI]
PTHrP total amount (pg/site)	4.26 ± 2.96 ^a^	12.45 ± 5.77 ^b^	19.67 ± 4.59 ^c^	12.13 ± 7.79	110.744	**<0.001 ^y^**	0.662 [0.529: 0.737]
PTHrP concentration (pg/µL)	17.68 (3.65:89.82)/32.11 ^a^	27.24 (0.42:101.27)/43.22 ^ab^	29.48 (14.92:61.15)/47.67 ^b^	26.06 (0.42:101.27)	6.263	**0.044 ^x^**	0.055 [0.000: 0.238]
RANKL total amount (pg/site)	51.4 (48.78:108.7)/40.2	51.4 (48.03:131.17)/41.37	51.78 (47.66:321.42)/41.43	51.4 (47.66:321.42)	0.047	0.977 ^x^	0.000 [-: -]
RANKL concentration (pg/µL)	223.49 (95.97:1449.35)/59.96 ^a^	121.50 (52.72:269.69)/37.59 ^b^	77.95 (49.20:680.57)/25.44 ^b^	130.34 (49.20:1449.35)	29.911	**<0.001 ^x^**	0.358 [0.148: 0.693]
OPG total amount (pg/site)	53.36 (44.11:71.87)/56.72 ^a^	46.7 (41.15:59.65)/33.3 ^b^	46.7 (39.3:58.17)/32.98 ^b^	49.29 (39.3:71.87)	18.109	**<0.001 ^x^**	0.207 [0.058: 0.480]
OPG concentration (pg/µL)	227.18 (91.94:920.95)/64.22 ^a^	104.63 (48.28:260.36)/38.13 ^b^	72.98 (44.91:140.53)/20.65 ^c^	107.66 (44.91:920.95)	46.913	**<0.001 ^x^**	0.576 [0.296: 0.980]
RANKL/OPG ratio	0.98 (0.72:1.88)/23.61 ^a^	1.1 (0.93:2.71)/48.81 ^b^	1.14 (0.94:7.23)/50.57 ^b^	1.08 (0.72:7.23)	22.201	**<0.001 ^x^**	0.259 [0.087: 0.555]

Values are median (minimum:maximum)/mean rank, except for PTHrP total amount, which is presented as mean ± SD. Total amount is the mean amount recovered per sampled site (pg/site); concentration is total amount divided by GCF volume per site (pg/μL); the RANKL/OPG ratio was calculated from total amounts. ^x^ Kruskal–Wallis H test; ^y^ Welch’s ANOVA. ^a–c^ Within a row, values with different superscript letters differ significantly (*p* < 0.05), whereas values sharing a letter do not. ES [95% CI], effect size with 95% confidence interval: rank-based eta-squared (η^2^H) for the Kruskal–Wallis test and eta-squared (η^2^) for Welch’s ANOVA. Bold *p* values indicate statistical significance (*p* < 0.05). GCF, gingival crevicular fluid; OPG, osteoprotegerin; PTHrP, parathyroid hormone-related protein; RANKL, receptor activator of nuclear factor kappa B ligand; SD, standard deviation.

**Table 4:** Covariate-adjusted comparisons of GCF PTHrP, RANKL and OPG levels and the RANKL/OPG ratio in the study groups.

Outcome	Adjusted Mean [95% CI]			Difference in Adjusted Means [95% CI]			Test Statistic	*p*
	Periodontal Health (*n* = 27)	Gingivitis (*n* = 27)	Periodontitis (*n* = 27)	Gingivitis vs. Health	Periodontitis vs. Health	Periodontitis vs. Gingivitis		
PTHrP total amount (pg/site) adjusted for age and sex	4.67 ^a^ [2.83: 6.52]	12.57 ^b^ [10.71: 14.43]	19.08 ^c^ [17.12: 21.05]	7.90 [4.78: 11.02]	14.41 [10.95: 17.86]	6.51 [2.92: 10.10]	57.27	**<0.001**
PTHrP total amount (pg/site) adjusted for GCF volume	5.09 ^a^ [2.87: 7.31]	12.40 ^b^ [10.64: 14.15]	18.90 ^c^ [16.73: 21.06]	7.30 [3.78: 10.83]	13.80 [9.35: 18.26]	6.50 [3.12: 9.88]	28.84	**<0.001**
RANKL total amount (pg/site) adjusted for age and sex	56.74 [50.57: 62.91]	62.65 [51.90: 73.41]	71.60 [51.62: 91.58]	NA	NA	NA	1.50	0.230
OPG total amount (pg/site) adjusted for age and sex	53.05 ^a^ [50.89: 55.21]	47.65 ^b^ [45.48: 49.83]	48.04 ^b^ [45.74: 50.33]	−5.39 [−9.04: −1.75]	−5.01 [−9.05: −0.97]	0.38 [−3.82: 4.58]	8.03	**<0.001**
RANKL/OPG ratio adjusted for age and sex	1.08 ^a^ [0.95: 1.20]	1.31 ^a^ [1.09: 1.53]	1.49 ^a^ [1.10: 1.88]	0.23 [−0.01: 0.47]	0.41 [−0.07: 0.90]	0.18 [−0.34: 0.70]	4.48	**0.015**

Values are adjusted means [95% CI] or differences in adjusted means [95% CI]. Models adjusted for age and sex included group and sex as fixed factors, their interaction, and age as a covariate; the PTHrP model adjusted for GCF volume included GCF volume per site as a covariate. Estimation used HC3 heteroscedasticity-consistent standard errors (PTHrP), bootstrap resampling with 5000 samples (RANKL, RANKL/OPG ratio), or ordinary least squares (OPG). Test statistic: F for the group effect, with 2 and 74 degrees of freedom for models adjusted for age and sex and 2 and 77 degrees of freedom for the model adjusted for GCF volume. ^a–c^ Within a row, adjusted means with different superscript letters differ significantly (Bonferroni-adjusted *p* < 0.05), whereas means sharing a letter do not. Age was not significantly associated with any outcome (PTHrP, *p* = 0.510; RANKL, *p* = 0.133; OPG, *p* = 0.417; RANKL/OPG ratio, *p* = 0.144). Group × sex interaction: PTHrP, *p* = 0.247; RANKL, *p* = 0.045; OPG, *p* = 0.139; RANKL/OPG ratio, *p* = 0.045. Bold values indicate statistical significance (*p* < 0.05). NA, not applicable (no significant group effect); CI, confidence interval; GCF, gingival crevicular fluid; PTHrP, parathyroid hormone-related protein; RANKL, receptor activator of nuclear factor kappa B ligand; OPG, osteoprotegerin.

**Table 5:** Correlations between GCF biomarker total amounts, the RANKL/OPG ratio, GCF volume and clinical periodontal parameters.

	PTHrP Total Amount		RANKL Total Amount		OPG Total Amount		RANKL/OPG Ratio	
	*r*	Adjusted *p*	*r*	Adjusted *p*	*r*	Adjusted *p*	*r*	Adjusted *p*
PTHrP total amount								
RANKL total amount	−0.077 ^x^	0.583						
OPG total amount	−0.530 ^x^	**<0.001**	0.520 ^x^	**<0.001**				
RANKL/OPG ratio	0.481 ^x^	**<0.001**	0.147 ^x^	0.246	−0.633 ^x^	**<0.001**		
GCF volume per site (µL/site)	0.647 ^x^	**<0.001**	0.030 ^x^	0.857	−0.352 ^y^	**0.001**	0.389 ^x^	**<0.001**
Probing depth (mm)	0.576 ^x^	**<0.001**	0.086 ^x^	0.548	−0.191 ^x^	0.122	0.291 ^x^	**0.013**
CAL (mm)	0.658 ^x^	**<0.001**	0.012 ^x^	0.914	−0.206 ^x^	0.093	0.229 ^x^	0.061
Plaque index	0.663 ^x^	**<0.001**	−0.058 ^x^	0.687	−0.434 ^x^	**<0.001**	0.438 ^x^	**<0.001**
Gingival index	0.648 ^x^	**<0.001**	0.021 ^x^	0.889	−0.409 ^x^	**<0.001**	0.423 ^x^	**<0.001**

^x^ Spearman’s rho correlation; ^y^ Pearson correlation. *p*-values were adjusted for multiple testing using the Benjamini–Hochberg false discovery rate procedure (26 tests). Bold values indicate statistical significance (adjusted *p* < 0.05). CAL, clinical attachment level; GCF, gingival crevicular fluid; PTHrP, parathyroid hormone-related protein; RANKL, receptor activator of nuclear factor kappa B ligand; OPG, osteoprotegerin.

## Data Availability

The data supporting the findings of this study are not publicly available due to privacy and ethical restrictions, but may be made available from the corresponding author upon reasonable request and with permission from the relevant institutional authorities.

## Abbreviations

The following abbreviations are used in this manuscript:

ANOVA	analysis of variance
BOP	bleeding on probing
CAL	clinical attachment level
CI	confidence interval
ELISA	enzyme-linked immunosorbent assay
GCF	gingival crevicular fluid
GI	gingival index
HBV	hepatitis B virus
HC3	heteroscedasticity-consistent standard errors, type 3
HIV	human immunodeficiency virus
HRP	horseradish peroxidase
ICC	intraclass correlation coefficient
IL-1β	interleukin-1 beta
IL-6	interleukin-6
mRNA	messenger ribonucleic acid
OPG	osteoprotegerin
PBS	phosphate-buffered saline
PD	probing depth
PI	plaque index
PTH	parathyroid hormone
PTH1R	parathyroid hormone/parathyroid hormone-related protein type 1 receptor
PTHrP	parathyroid hormone-related protein
RANK	receptor activator of nuclear factor kappa B
RANKL	receptor activator of nuclear factor kappa B ligand
RBL	radiographic bone loss
SD	standard deviation
STROBE	Strengthening the Reporting of Observational Studies in Epidemiology
TMB	3,3′,5,5′-tetramethylbenzidine
TNF-α	tumor necrosis factor alpha
